# Vitamin D Supplementation: Oxidative Stress Modulation in a Mouse Model of Ovalbumin-Induced Acute Asthmatic Airway Inflammation

**DOI:** 10.3390/ijms22137089

**Published:** 2021-06-30

**Authors:** Teodora-Irina Adam-Bonci, Eduard-Alexandru Bonci, Alina-Elena Pârvu, Andrei-Ioan Herdean, Augustin Moț, Marian Taulescu, Andrei Ungur, Raluca-Maria Pop, Corina Bocșan, Alexandru Irimie

**Affiliations:** 1 Department of Pathophysiology, “Iuliu Hațieganu” University of Medicine and Pharmacy, 400012 Cluj-Napoca, Romania; teadaria@gmail.com (T.-I.A.-B.); parvualinaelena@yahoo.com (A.-E.P.); 2 Department of Oncological Surgery and Gynecologic Oncology, “Iuliu Hațieganu” University of Medicine and Pharmacy, 400015 Cluj-Napoca, Romania; airimie@umfcluj.ro; 3 Department of Anatomy and Embryology, “Iuliu Hațieganu” University of Medicine and Pharmacy, 400006 Cluj-Napoca, Romania; dr.herdean.andrei@gmail.com; 4 Department of Chemistry, Faculty of Chemistry and Chemical Engineering, “Babeș-Bolyai” University, 400028 Cluj-Napoca, Romania; augustin.mot@ubbcluj.ro; 5 Department of Pathology, Faculty of Veterinary Medicine, University of Agricultural Sciences and Veterinary Medicine Cluj-Napoca, 400372 Cluj-Napoca, Romania; marian.taulescu@usamvcluj.ro (M.T.); andrei.ungur@usamvcluj.ro (A.U.); 6 Synevovet Laboratory, 81 Pache Protopopescu, 021408 Bucharest, Romania; 7 Department of Pharmacology, Toxicology and Clinical Pharmacology, “Iuliu Hațieganu” University of Medicine and Pharmacy, 400337 Cluj-Napoca, Romania; raluca_parlog@yahoo.com (R.-M.P.); corinabocsan@yahoo.com (C.B.)

**Keywords:** oxidative stress, vitamin D, asthma, inflammation

## Abstract

Asthma oxidative stress disturbances seem to enable supplementary proinflammatory pathways, thus contributing to disease development and severity. The current study analyzed the impact of two types of oral vitamin D (VD) supplementation regimens on the redox balance using a murine model of acute ovalbumin-induced (OVA-induced) asthmatic inflammation. The experimental prevention group received a long-term daily dose of 50 µg/kg (total dose of 1300 µg/kg), whereas the rescue group underwent a short-term daily dose of 100 µg/kg (total dose of 400 µg/kg). The following oxidative stress parameters were analyzed in serum, bronchoalveolar lavage fluid (BALF) and lung tissue homogenate (LTH): total oxidative status, total antioxidant response, oxidative stress index, malondialdehyde and total thiols. Results showed that VD significantly reduced oxidative forces and increased the antioxidant capacity in the serum and LTH of treated mice. There was no statistically significant difference between the two types of VD supplementation. VD also exhibited an anti-inflammatory effect in all treated mice, reducing nitric oxide formation in serum and the expression of nuclear factor kappa B p65 in the lung. In conclusion, VD supplementation seems to exhibit a protective role in oxidative stress processes related to OVA-induced acute airway inflammation.

## 1. Introduction

Asthma is a chronic inflammatory condition of the airways [1], triggered and sustained by genetic conditioning, environmental factors and possibly some degree of variation in microbiome and metabolite balance [2]. The prevalence of bronchial asthma [3] has been steadily increasing over the past years [4], notably in the pediatric segment [5], and many of these patients evolve into adults who have asthma [6]. Considering the significant burden generated by disease management and symptom control, considerable research has been conducted recently in the field of adjuvants for classical asthma therapy. One such topic evolves around the benefit of vitamin D (VD) supplementation, with more data required to support the intervention, as mentioned in the Global Initiative for Asthma Guideline [7]. 

The pathophysiology of asthma is well described, with the vast majority of asthmatics displaying an imbalanced immune reaction, and with a prevalence of T-helper 2 (Th2) immune reply [8]. This response is associated with a particular profile of cytokines, such as interleukins 4, 5 and 13 (IL-4, IL-5, IL-13) [9], that not only amplify inflammation, but also contribute to an increase in oxidant forces [10]. 

Considerable research has acknowledged the role of oxidative stress in asthma pathogenesis. This phenomenon occurs due to the imbalance between the protective antioxidant systems of the lung and the processes that generate oxidizing products, resulting in more of the latter. Under normal conditions, antioxidants reestablish redox homeostasis. In asthma, increased oxidizing stimuli are encountered, which amplify a vicious circle in the inflammatory-immune response of asthma [11]. These triggers are sourced both externally, such as environmental allergens, pollution, or cigarette smoke, and internally, produced at the cellular level. The cells recruited in the asthmatic immune-inflammatory cascade produce reactive oxygen species (ROS) [12]. This process increments the amount of oxidizing products, consequently activating redox-responsive intracellular signaling mechanisms. One such mechanism involves the activation of the nuclear factor kappa B (NF-κB) pathway [13], thus complementing the vicious circle that increases the expression of inflammatory molecules [14].

Serum VD status has been intensely investigated in relation to asthma severity, both adult [15] and pediatric [16,17]. The literature specifies several adverse disease outcomes in case of VD deficiency, such as poor symptom management [18] with severe asthma exacerbations [19], decreased lung function [20] and a modified response to inhaled corticosteroid therapy [21]. Studies following the clinical benefits of VD supplementation reported a reduced incidence of asthma attacks [22], including severe exacerbations [23]. Positive results also noted an improved performance on spirometry tests [22], benefiting adult asthmatics smokers [24], whereas increased serum levels of VD correlated with adequate pulmonary function and good quality of life in pediatric asthmatics [25]. Other favorable outcomes included a decreased number of asthma attacks treated with systemic corticosteroids [26]. Thus, the body of evidence points to VD playing a potentially noteworthy role in the clinical management of asthma [27], even though some report conflicting data [28]. VD supplementation is a relatively cheap, easy and non-invasive intervention in asthma management. To fundament the gains of such a recommendation, more studies are needed to explain why VD exerts the expected beneficial actions in asthma [29]. 

Bronchial asthma is a disorder comprising several phenotypes in pediatric patients [30] and adults [31]. Over the last three decades, various animal models of asthma were developed to research the disease mechanisms, progression and effects of various therapeutic agents [32]. Out of all species, the mouse is the one most resorted to in experiments. Ovalbumin (OVA), an allergen derived from chicken eggs, with aluminum hydroxide as an adjuvant, is recognized as an approach to induce a type-2 inflammation [33]. The ovalbumin model corresponds to eosinophilic asthma in humans [34], one of the most frequently encountered clinical phenotypes. Moreover, *BALB*/*c* mice lacking a vitamin D receptor did not develop airway hyperresponsiveness [35], distinguishing this species as suitable for an experiment involving VD supplementation. Although oxidative stress is acknowledged to play an essential role in the evolution of asthma, influencing clinical outcomes and disease control, few researchers have studied the relationship between redox balance and VD status, but with results showing a potential protective effect [36].

The present study investigated the effects of oral VD supplementation in oxidative stress modulation, using a murine model of induced acute asthmatic lung inflammation.

## 2. Results

### 2.1. Oxidative Stress

#### 2.1.1. Serum

**Figure 1:**
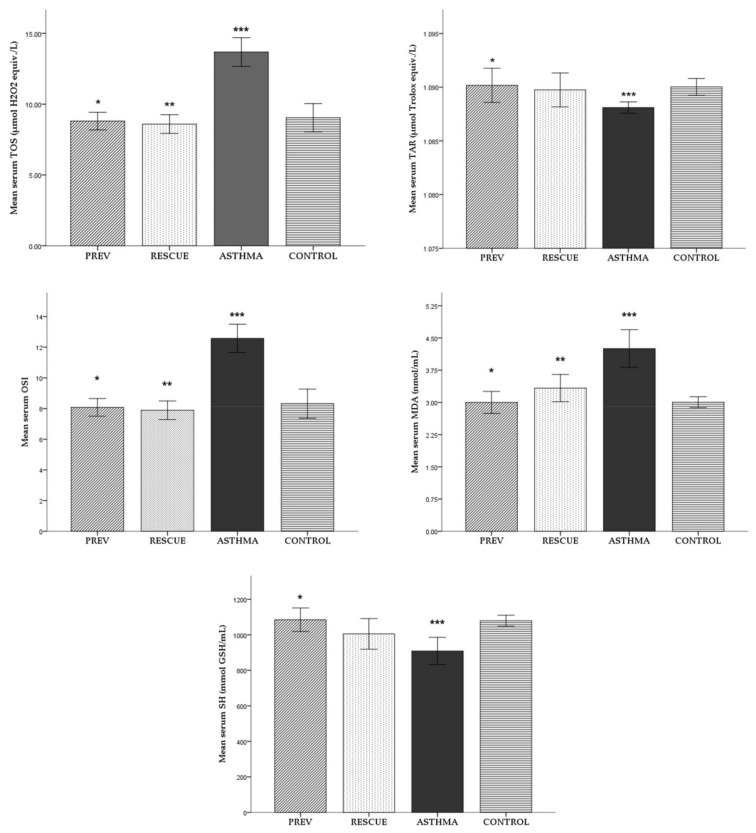
Mean levels (± standard deviation) of oxidative stress parameters in serum. Comparison between experimental groups: * *p* < 0.05 PREV versus ASTHMA group; ** *p* < 0.05 RESCUE versus ASTHMA group; *** *p* < 0.05 ASTHMA versus CONTROL. Abbreviations: TOS = total oxidative status, TAR = total antioxidant response, OSI = oxidative stress index, MDA = malondialdehyde, SH = total thiols; PREV = long-term low-dose vitamin D supplementation, RESCUE = short-term high-dose vitamin D supplementation, ASTHMA = positive control, CONTROL = negative control.

For each experimental group, the mean serum values (± standard deviation) of the explored oxidative stress parameters are reported in Figure 1. The averages of the studied parameters were compared between the experimental groups, with the corresponding level of statistical significance noted in Figure 1.

The level of oxidative stress was increased in the experimental asthma group (ASTHMA), with total oxidative status (TOS), oxidative stress index (OSI) and malondialdehyde (MDA) parameters significantly elevated compared to the negative control group (CONTROL). The antioxidant capacity decreased in the ASTHMA lot, with a significantly lower total antioxidant response (TAR) level than CONTROL. Consistently, in opposition to the CONTROL group, the level of serum total thiols (SH) was significantly reduced by the experimental inflammation induced in the ASTHMA group.

Preventive therapy, defined as long-term low-dose VD supplementation (PREV group), and rescue therapy with short-term high-dose VD (RESCUE group) reduced the level of oxidative stress in serum: in both groups, TOS, MDA and OSI were significantly lower compared to ASTHMA. However, only preventive VD therapy seemed to have a significant role in the antioxidant response, with TAR elevated in the PREV group. VD supplementation increased SH levels, but only in PREV group did it show a statistically significant antioxidant effect. 

VD supplementation seemed to improve the redox balance better when administered in a long-term low dose. However, we did not observe any statistically significant differences between the two treated groups.

#### 2.1.2. Broncho-Alveolar Lavage Fluid (BALF)

**Figure 2:**
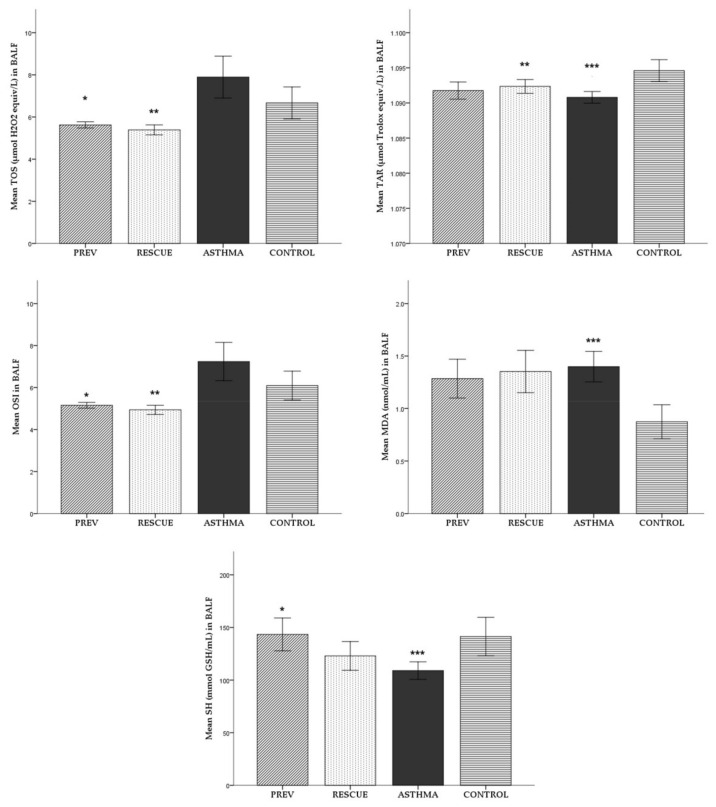
Mean levels (± standard deviation) of oxidative stress parameters in BALF. Comparison between experimental groups: * *p* < 0.05 PREV versus ASTHMA group; ** *p* < 0.05 RESCUE versus ASTHMA group; *** *p* < 0.05 ASTHMA versus CONTROL. Abbreviations: BALF = broncho-alveolar lavage fluid, TOS = total oxidative status, TAR = total antioxidant response, OSI = oxidative stress index, MDA = malondialdehyde, SH = total thiols; PREV = long-term low-dose vitamin D supplementation, RESCUE = short-term high-dose vitamin D supplementation, ASTHMA = positive control, CONTROL = negative control.

For each experimental group, the average (± standard deviation) of BALF oxidative stress parameters is reported in Figure 2. The mean values of the studied parameters were compared between the experimental groups, with the corresponding level of statistical significance noted in Figure 2.

We observed a surge in oxidative stress in BALF, marked by higher levels of TOS, OSI and MDA in the ASTHMA group than in the CONTROL group. However, only MDA proved to be significantly elevated. Consistently, a TAR measurement showed that the antioxidant response was significantly lower in the ASTHMA group than in the inflammation-free CONTROL group. 

Both preventive and rescue VD therapy reduced the degree of oxidative stress in BALF, decreasing TOS, MDA and OSI compared to ASTHMA. Even so, in the case of both treated groups, the observed reduction in oxidation was statistically significant only for TOS and OSI. Regarding the antioxidant response in BALF, the short-term high-dose therapy with VD administered in the RESCUE group significantly increased TAR level compared to ASTHMA.

In contrast to the CONTROL group, the SH level in BALF was significantly reduced by the inflammation in the ASTHMA group. VD supplementation seemed to produce an antioxidant effect by increasing the SH level in mice with induced inflammation from the PREV and RESCUE groups. Even so, only the long-term low-dose regimen performed in the PREV group produced a statistically significant improvement.

Except for TOS and TAR, VD supplementation seemed to improve oxidative stress when administered in a long-term low-dose. However, we did not observe statistically significant differences between the two treated groups.

#### 2.1.3. Lung Tissue Homogenate (LTH)

**Figure 3:**
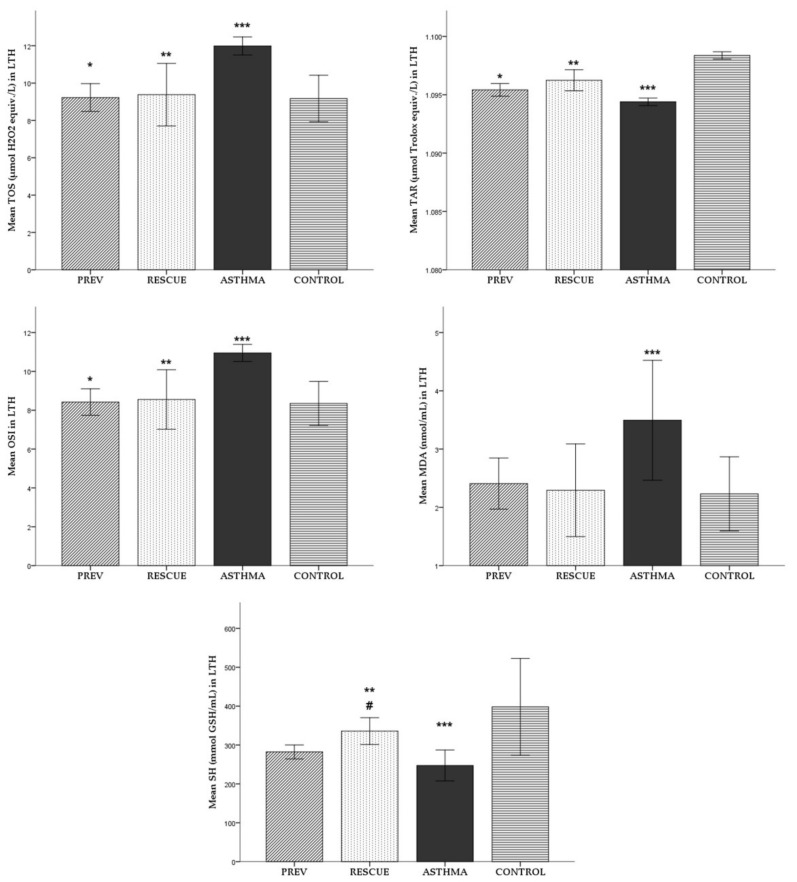
Mean levels (± standard deviation) of oxidative stress parameters in LTH. Comparison between experimental groups: * *p* < 0.05 PREV versus ASTHMA group; ** *p* < 0.05 RESCUE versus ASTHMA group; *** *p* < 0.05 ASTHMA versus CONTROL; # *p* < 0.05 PREV versus RESCUE. Abbreviations: LTH = lung tissue homogenate, TOS = total oxidative status, TAR = total antioxidant response, OSI = oxidative stress index, MDA = malondialdehyde, SH = total thiols; PREV = long-term low-dose vitamin D supplementation, RESCUE = short-term high-dose vitamin D supplementation, ASTHMA = positive control, CONTROL = negative control.

For each experimental group, the average (± standard deviation) of oxidative stress parameters is reported in Figure 3. The mean values of the studied parameters were compared between the experimental groups, with the corresponding level of statistical significance noted in Figure 3.

The ASTHMA group had statistically significant higher levels of TOS, OSI and MDA in LTH compared to CONTROL. The TAR level was significantly lower in the ASTHMA group in contrast to the CONTROL group. 

VD supplementation reduced the level of TOS, MDA and OSI in both the PREV and RESCUE groups, compared to the level of oxidative stress in ASTHMA, with statistically significant results for TOS and OSI, but not for MDA. VD therapy had a significant antioxidant effect, leading to increased TAR levels in both PREV and RESCUE, as opposed to the TAR status in ASTHMA.

Due to the induced inflammation, the SH level was statistically significantly lower in ASTHMA than in the CONTROL group. VD therapy increased the SH level in the treated asthmatic mice from the PREV and RESCUE lots compared to ASTHMA. However, only in the RESCUE group that received short-term high-dose VD was the observed benefic effect in the SH level increase statistically significant. Moreover, the mean SH value in the RESCUE group was significantly higher than the mean SH in the PREV group.

### 2.2. Inflammation

#### 2.2.1. Inflammatory Cytokines

**Figure 4:**
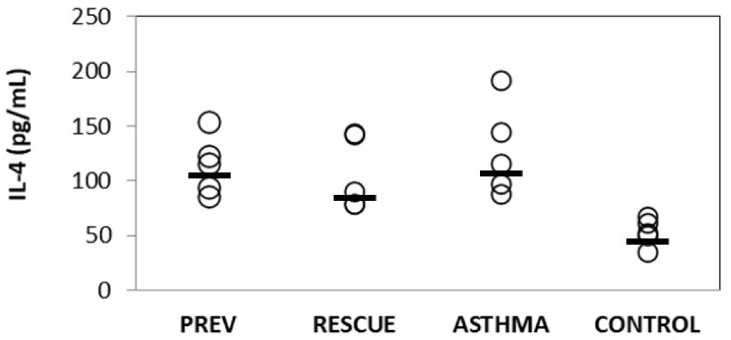
Variation of IL-4 levels in broncho-alveolar lavage fluid (Abbreviations: IL-4 = interleukin 4; PREV = long-term low-dose vitamin D supplementation, RESCUE = short-term high-dose vitamin D supplementation, ASTHMA = positive control, CONTROL = negative control. Note: circles = individual values; horizontal line = median.).

To assess the degree of induced inflammation in asthmatic mice and investigate the anti-inflammatory role of VD supplementation, we determined the IL-4 level from BALF. The results are illustrated in Figure 4, as individual values and the corresponding group median. The experimental inflammation significantly increased IL-4 in BALF from mice in the ASTHMA group compared to the CONTROL group (*p* < 0.05). VD treatment had an anti-inflammatory effect, producing a decrease in the BALF level of IL-4 in mice from PREV and RESCUE groups in opposition to the ASTHMA group, but the observed differences were not statistically significant.

#### 2.2.2. Nitric Oxide 

**Figure 5:**
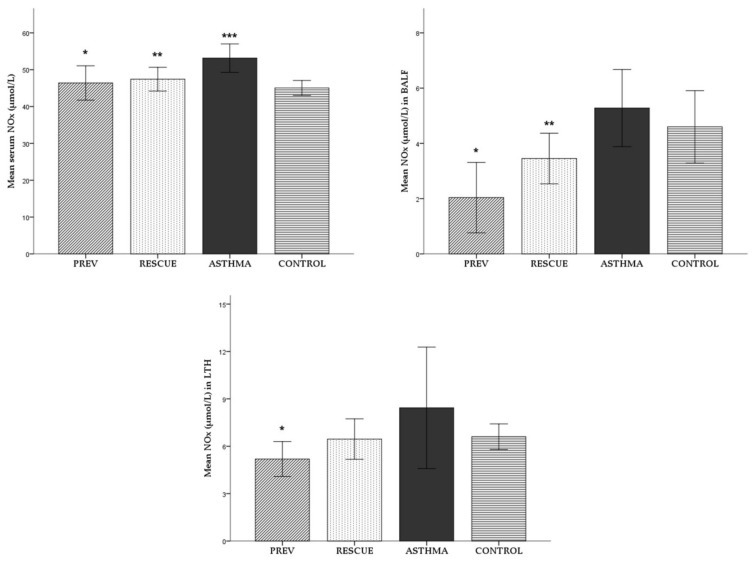
NOx levels in serum, BALF and LTH. Values are expressed as mean ± SD. Comparison between experimental groups: * *p* < 0.05 PREV versus ASTHMA group; ** *p* < 0.05 RESCUE versus ASTHMA group; *** *p* < 0.05 ASTHMA versus CONTROL. Abbreviations: BALF = broncho-alveolar lavage fluid; LTH = lung tissue homogenate; NOx = level of inorganic nitrites and nitrates; PREV = long-term low-dose vitamin D supplementation, RESCUE = short-term high-dose vitamin D supplementation, ASTHMA = positive control, CONTROL = negative control. Note: a Mann–Whitney test was applied for the analysis of LTH NOx values.

The serum, BALF and LTH levels of inorganic nitrites and nitrates (NOx) and the stable end metabolites of nitric oxide (NO) were assessed to evaluate the NO synthesis. To evaluate the degree of inflammation and the effect of VD supplementation, NOx was assessed as a proinflammatory marker. The mean levels (± standard deviation) in serum, BALF and LTH are recorded in Figure 5.

The NOx levels in all biological samples were higher in mice from the ASTHMA group. However, a statistically significant increase was observed only for serum NOx in ASTHMA mice compared to CONTROL (*p* < 0.05), as seen in Figure 5. 

In both the PREV and RESCUE groups, VD supplementation produced an anti-inflammatory effect. In PREV group we observed a sharp decrease of NOx in serum, BALF and LTH compared to ASTHMA (*p* < 0.05). Regarding the RESCUE group, the short-term high-dose VD therapy induced a decrease in the NOx level in all types of samples but with statistical significance only in serum and BALF (*p* < 0.05).

### 2.3. Pathology

**Figure 6:**
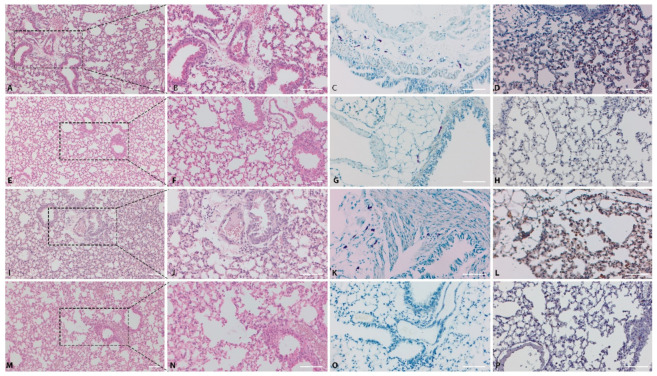
Histological and immunohistochemical findings of lungs from experimental mice groups. PREV Group: histological changes of pulmonary parenchyma, HE stain (**A**,**B**); TB staining showing numerous peribronchiolar mast cells (**C**); NF-kB p65 immune expression in the lungs, IHC stain (**D**). RESCUE group: Lung, HE stain (**E**,**F**) and TB stain (**G**); NF-kB p65 immunoexpression in the lungs, IHC stain (**H**). ASTHMA group: Lung, HE stain (**I**,**J**) and TB stain (**K**); NF-kB p65 immunoexpression in the lungs, IHC stain (**L**). CONTROL group: Lung, HE stain (**M**,**N**) and TB stain (**O**); NF-kB p65 immunoexpression in the lungs, IHC stain (**P**). Bar = 50um. Abbreviations: HE = hematoxylin-eosin, TB = toluidine blue, NF-kB p65 = nuclear factor kappa B p65, IHC = immunohistochemical staining, PREV = long-term low-dose vitamin D supplementation, RESCUE = short-term high-dose vitamin D supplementation, ASTHMA = positive control, CONTROL = negative control.

In the CONTROL group, no essential microscopic changes were observed in hematoxylin-eosin (HE) stained slides (Figure 6M,N). The toluidine blue (TB) reaction was negative in all examined fields (Figure 6O). The nuclear factor kappa B p65 (NF-kB p65) expression was cytoplasmic and minimal, represented by scattered positive cells within the pulmonary parenchyma (Figure 6P). 

In the ASTHMA group, the peribronchiolar area and alveolar septa were multifocally infiltrated with aggregates of small lymphocytes and macrophages (Figure 6I,J). Numerous TB positive cells (mast cells) were identified in the peribronchiolar area in all individuals from the ASTHMA group (Figure 6K). The number and intensity of NF-kB p65 positive cells were significantly higher (*p*< 0.05) in ASTHMA (Figure 6L) compared to CONTROL (Figure 6P).

In the PREV group, treated with a long-course low-dose of VD, the peribronchial and perivascular areas were mildly infiltrated with mononuclear cells, predominated by small lymphocytes and macrophages, associated with mild interstitial edema (Figure 6A,B). The grade of inflammatory reaction was significantly lower in the PREV group than in ASTHMA (*p* < 0.05). Furthermore, the number of TB positive cells (mast cells) in the PREV group (Figure 6C) was lower compared to the untreated ASTHMA group (*p* = 0.7). 

In the RESCUE group, treated with short-course high-dose VD, the histological, histochemical and immunohistochemical findings were similar to those found in the PREV group. Compared to the ASTHMA group, only scattered inflammatory cells (Figure 6E,F) were identified in the peribronchiolar area, with the inflammatory score significantly lower than in ASTHMA (*p* < 0.05). Similarly, fewer TB positive cells (Figure 6G) were noticed (*p* = 0.12).

Regarding the NF-kB p65 expression in the VD treated groups: compared to the ASTHMA group (Figure 6L), both the PREV (Figure 6D) and RESCUE (Figure 6H) groups presented significantly lower intensity scores (*p* < 0.05) and fewer NF-kB p65 immunopositive cells (*p* < 0.05). Even though the inflammatory score, the mean number of TB positive cells and the NF-kB p65 expression scores were lower in the PREV group than in RESCUE, the observed differences were not statistically significant.

The periodic acid-Schiff/Alcian blue (PAS-AB) staining was negative in all groups, revealing that the mice in this experiment did not present an increase in airway goblet cells.

## 3. Discussion

The medical literature recognizes the notable role of VD in bronchial asthma [27,37]. Numerous studies already presented a potential link between VD deficiency status and various poor asthma outcomes. Interventional studies explored and described the protective role of VD supplementation in various conditions: prenatally, to prevent asthma in offspring [38] or in adults [39], and pediatric [40] disease control. Trials on this topic are still ongoing [41]. Results generally point toward a positive effect of VD in asthma management. However, the body of data is heterogeneous: it is provided by various types of studies (observational, interventional) [42], some of which reported conflicting results, failing to support the expected beneficial outcome [43]. For this reason, the research into the mechanisms behind the benefic effects continues to be a subject of interest. 

Although accepted as pivotal in disease pathogenesis, oxidative stress modulation in asthma is a mechanism that was less studied in the context of VD treatment. For this reason, in the current study, we investigated how oral VD supplementation influenced the redox balance, using an animal model of induced acute asthmatic inflammation.

The element of novelty was provided by the oxidative stress evaluation in three biological products: serum, BALF and LTH. Compared to healthy controls, the experimental asthmatic inflammation produced a significant augmentation in oxidative forces (measured indirectly via TOS) and decreased the antioxidant capacity (assessed via TAR). The OSI, defined as the ratio of TOS to TAR, a measure of the extent of oxidative stress, was also increased in the asthmatic animal model.

However, in our study, the element of interest was VD supplementation on the oxidative stress associated with asthmatic inflammation. We observed that VD therapy significantly improved the redox balance in treated asthmatic mice compared to non-treated asthmatic subjects. Redox balance was improved by VD supplementation, as reflected by a significant decrease in OSI in serum, BALF and LTH. However, the protective effect of VD seems not to be influenced by the regimen type, since there was no statistical difference between the low-dose long-term and high-dose short-term regimens.

ROS are the main components of oxidative stress in bronchial asthma. These reactive species are produced by the airway epithelial cells and inflammatory cells recruited locally as part of the immune response: superoxide anion, hydroxyl radical and hydrogen peroxide [44]. To report the oxidative stress, we used the technique described by Erel [45] to determine TOS. This method quantifies the amount of oxidized iron that results from ROS catalyzed reactions. In our study, the experimental asthmatic inflammation determined a significant TOS elevation in serum and LTH compared to healthy mice. However, this increase was only of marginal statistical significance in BALF. Clinical studies reported similar findings: serum TOS was not only increased in pediatric asthmatics compared with healthy peers [46,47], but was also higher in the case of uncontrolled and partly controlled asthma compared with stable asthma [48]. TOS was inversely correlated with a peak expiratory flow rate [49], reportedly described as a marker of disease severity. Furthermore, DNA damage in peripheral lymphocytes was correlated with TOS in children with asthma [46]. However, in a mouse model of chronic OVA-induced asthmatic airway inflammation, VD treatment did not significantly modify the prooxidant–antioxidant balance [50], considered to be an indicator of acute oxidative stress. In contrast, in our study of acute asthmatic inflammation, both regimens of VD supplementation produced a significant TOS decrease in the serum, BALF and LTH of treated mice. The observed protective effect of VD on oxidant forces is consistent with existing data in the literature. One study reported that oral VD nanoemulsion therapy decreased oxidant species’ activity in the serum of mice with airway inflammation [51]. In another study, VD decreased ROS when added to human bronchial epithelial cell culture, thus alleviating lipopolysaccharide-induced oxidative stress [52]. 

MDA is considered an indirect marker of oxidative stress, a peroxidation product resulting from ROS action on lipids. MDA was identified in plasma, sputum, BALF [10] and exhaled breath condensate [53] from asthma patients. This aldehyde is an indicator of the disintegration of polyunsaturated fats and induces adverse effects on the protein and DNA structure [54]. Concerning asthma, the MDA level increased in the serum of patients, both adults [55] and children [56]. Experimental models of asthma in mice with OVA-induced inflammation showed that MDA was elevated in LTH [57,58] and BALF [59] compared to healthy animals. Similar to the previously published results, in our experiment, OVA-mediated asthmatic airway inflammation in mice triggered significantly higher MDA levels in serum, BALF and LTH than in negative controls. Both types of VD supplementation produced a benefic effect, decreasing peroxidation: MDA levels were significantly lower in the serum of treated mice, but the protective outcome was not statistically relevant in BALF and LTH. VD was administered orally, this route potentially influencing the bioavailability of the vitamin. Several previous models of chronic OVA-airway inflammation showed that parenteral VD therapy did produce a significant protective effect, decreasing MDA in LTH [50,60]. Therefore, our results might be influenced by the acute nature of the inflammation and the route of VD therapy. 

Physiologically, the lung is defended by enzymes such as superoxide dismutase (SOD), catalase and glutathione peroxidase (GSH-Px), which mediate antioxidant reactions [14]. In pathological conditions, such as bronchial asthma, ROS and reactive nitrogen species (RNS) overthrow the redox balance, thus rendering the protection ineffective [61]. What is worse, acute asthma attacks are associated with a specific decrease in antioxidant activity [62]. To observe if VD supplementation produced a protective effect on redox balance, we used the method of Erel [63] to report TAR. The technique measured the capacity of the analyzed sample to inhibit oxidant reactions mediated by the hydroxyl radical. In our model, TAR was significantly lower in serum from mice with induced asthmatic inflammation than from healthy controls. This finding is similar to other data reporting that pediatric [47] and adult [55] patients with asthmatic inflammation had significantly lower serum TAR compared to healthy controls. Even more, TAR was decreased in patients with uncontrolled asthma [64] and acute asthma exacerbations [65]. We also found that TAR was significantly decreased in the BALF and LTH of asthmatic mice compared to healthy subjects.

Similarly, the literature describes a cutback phenomenon in the antioxidant defense system of asthmatics. Catalase action was decreased in BALF from asthmatics, and SOD had lower activity in BALF and airway epithelial cells from both human and murine models of asthma [62,66]. Furthermore, transgenic mice that overexpressed SOD presented a lower susceptibility to developing allergen-induced airway alterations [67]. Previous research reported that serum TAR positively correlated with VD serum level in pediatric asthmatics [49]. Our results show that serum TAR benefited more from long-term low-dose VD therapy. Over a more extended period, VD supplementation would have higher chances of increasing the serum level, probably explaining our observed protective effect on serum TAR. Moreover, our findings show that TAR in LTH was improved by VD regardless of regimen, whereas only high-dose short-term VD supplementation significantly improved TAR level in BALF. An explanation might be provided by the timing of the high-dose VD regimen: in our experimental design, it was offered before the allergen challenge, the experimental equivalent of an acute asthma attack. Previous experiments showed that acute asthma exacerbations induced changes in the antioxidant forces, resulting in reduced glutathione levels in the exhaled breath condensate [68]. Thus, a high-dose VD intervention in the challenge step might have influenced the acute changes in redox balance favoring TAR. 

Sulphur containing compounds scavenge ROS, thus playing an antioxidant role [69]. The plasma pool of thiols is represented mainly by albumin and a smaller amount of low molecular weight thiols like cysteine or glutathione (GSH) [70]. The epithelium lining the airspace may be influenced by oxidative stress, leading to an increased local permeability and flow of plasma antioxidants into the epithelial lining fluid [71]. Even so, GSH is the most abundant antioxidant found both in the epithelial cells [10] and the fluid lining them [72]. Asthma is associated with a decrease in total thiols [55,73]. A mouse model of OVA-induced asthma showed a cellular loss of GSH [74]. ROS stimulated inflammatory cytokines, such as TGF-β1, consequently decreasing GSH by suppressing the expression of the enzymes involved in the synthesis [75]. In our experiment, mice with induced asthmatic inflammation presented a significantly lower number of total thiols in plasma, BALF and LTH than healthy controls. Thus, the oxidative stress associated with our induced inflammation seemed to decrease the pool of thiol antioxidants. VD therapy appeared to have a protective effect: mice that received the long-term low-dose VD regimen had higher levels of thiols in plasma and BALF, but not in LTH. However, during the challenge days (as part of the short-term high-dose regimen), VD supplementation led to a significant improvement of the total thiol pool in LTH, but not in BALF and serum. This result is consistent with data from the literature. GSH is mainly found inside cells and thus accounts for the majority of total thiols in LTH. A previous study showed that GSH significantly increased in cultured bronchial epithelial cells 24 hours after VD was added to the cell culture medium [76]. Moreover, VD supplementation was found to enhance the enzymes involved in GSH production [77]. Therefore, it seems that the role of short-term high-dose VD concerning the antioxidant system might be linked to intracellular GSH. 

In the pathogenesis of asthma, CD4+ T cells synthesize proinflammatory cytokines such as IL-4, IL-5 and IL-13 [78]. Firstly, these cytokines stimulate the B cell production of immunoglobulin E (IgE), further augmenting the activation of immune cells and signaling molecules involved in asthma development [79]. Secondly, they enhance oxidative stress [80], with IL-4 increasing ROS production via the activation of nicotinamide adenine dinucleotide phosphate oxidase [81]. Previous mouse models of both acute and chronic induced asthma inflammation presented elevated levels of IL-4 in BALF [82,83]. Our study also showed that IL-4 was significantly elevated in BALF from mice with induced inflammation compared to controls, thus verifying our model. The literature mentions that asthmatic mice that received an intraperitoneal VD injection presented a diminished Th2 cytokine response in BALF, including the IL-4 level [84]. In our experiment, oral VD therapy also seemed to have an anti-inflammatory effect: regardless of the regimen, treated mice displayed decreased IL-4, but the outcome did not pass the threshold of statistical significance. The serum status of VD was positively related to asthma severity [85] and the potency of VD protective effects [86]. A possible explanation for the less intense VD action observed in our study may be related to the route of administration: oral supplementation produces a nonlinear dose-dependent increase in serum VD [87]. 

Alongside the pathology lung inflammation score, NF-κB immunohistochemistry was used to assess the experimental model. The NF-κB signaling pathway is abnormally enabled in the airway epithelium of both human patients and animal models of asthma [88]. This activation leads to the recruitment of proinflammatory cytokines that expedite the local infiltration of leukocytes [89]. Previous studies using murine asthma models identified an increase in NF-κB activation and a positive correlation with oxidative stress parameters, such MDA [90]. Similarly, in our experiment, the immunohistochemical expression of NF-κB was increased in mice with induced asthmatic inflammation. Lung tissue sections from VD treated mice, regardless of the regimen, had significantly lower scores for inflammatory cell infiltration and lower NF-κB immunopositivity. Our findings support previous hypotheses placing NF-κB in correlation with the extent of oxidative stress processes in asthma [90].

NO is a molecule involved in signal transmission and plays a pivotal role in inflammation [91]. It may display a dual role: physiologically, it acts as a signal transducer and paracrine mediator, while excess NO and reactive nitrogen species (RNS) produce a cyto-destructive effect [92]. The amount of NO in the airway results from the balance between the enzymatic production via nitric oxide synthase (NOS) and its consumption in the reaction with other molecules [10]. There are three isoforms of NOS: two of them are constitutively expressed in tissues, the neuronal NOS (nNOS) and endothelial NOS (eNOS). The third isoform, theinducible NOS (iNOS) [91] is overexpressed in asthma [93]. In part, iNOS is also generated by proinflammatory cytokines, such as IL-4 [94]. Thus, NO levels are known to be increased in asthmatic airways [95]. For this reason, measuring the fraction of exhaled NO is a sensitive clinical indicator of airway inflammation and can be used to assess the anti-inflammatory response to steroid treatment [96]. NO reacts with oxygen or ROS to produce NOx (nitrite, NO_2_^-^ and nitrate, NO_3_^-^) and RNS [61]. NO_3_^-^ was increased in the BALF of asthmatics in both the acute and late phase of allergen challenge [95]. In our study, the experimental asthmatic inflammation produced a significant increase in NOx serum, but not in BALF and LTH. However, VD treatment induced a statistical decrease in NOx in all biological fluids. Thus, our findings might point toward an anti-inflammatory effect of VD. 

This study has several limitations. First of all, it used only one experimental model of asthma, which mimicked eosinophilic inflammation. Therefore, the modulatory effect of VD upon the oxidative stress generated by distinct pathophysiologic mechanisms could not be reported. Despite comparable clinical symptoms, distinct asthma endotypes may react differently to the same therapeutic interventions. Furthermore, most studies observed chronic asthma models, while our model reproduced a short-term inflammation. Therefore, the data we collected should be interpreted in the context of an acute allergen challenge. Secondly, we used a validated murine asthma model; we verified our experiment using a pathology analysis and a cytokine response, but a measurement of airway hyperresponsiveness was not done. However, in clinical practice, lung function tests play an essential role in assessing the clinical control of asthma. Finally, the literature reports that serum VD sufficiency status was associated with better asthma outcomes. For this reason, most human studies correlate the serum level of 25-hydroxyvitamin D (25-OH-VD) with various asthma characteristics. In our study, we did not check the serum level of 25-OH-VD of treated mice. Therefore, we could not report a relationship between the benefic antioxidant effects and VD sufficiency status or 25-OH-VD serum level. Further research could consider evaluating the 25-OH-VD serum level in mice before and after treatment, while considering the particular shorter half-life of 25-OH-VD specific to this species (1.4 days) [97,98] compared to humans (15 days) [99].

## 4. Materials and Methods

### 4.1. Experimental Design

#### 4.1.1. Animal Subjects

Female *BALB*/*c* mice aged 6–7 weeks, with a mean weight of 17.9 (± 0.34) g, were chosen to evaluate the role of VD supplementation on the development and severity of asthmatic lung inflammation and, respectively, the associated oxidative stress. The animals were housed in polypropylene cages, in a controlled laboratory environment (12 h light/dark cycle, at an ambiental temperature of 21°C), with restrictive natural light exposure and free access to water and standard pellet food (VD free). The experimental procedures in this study were done according to the national and international guidelines for the care and use of animals, followed the Helsinki Declaration on animal studies and received ethical approval from both “Iuliu Hațieganu” University of Medicine and Pharmacy Cluj-Napoca (nr. 163/20.05.2019) and the Romanian National Sanitary Veterinary and Food Safety Authority (nr. 167/29.05.2019). All experiments were conducted in triplicate.

#### 4.1.2. Experimental Protocol: Ovalbumin-Mediated Asthma Inflammation 

For the current study, we selected a validated model of acute murine asthma inflammation [100]. Ovalbumin (OVA, Grade V; Sigma Aldrich, Merck KGaA, Darmstadt, Germany) adsorbed on aluminum hydroxide (alum, Sigma Aldrich, Merck KGaA, Darmstadt, Germany) was freshly prepared as a 50% OVA-alum solution [100] and used for the sensitization step. An OVA with similar purity was prepared as a 40% solution [100], using a sterile saline solution (NaCl 0.9%, B. Braun Pharmaceuticals S.A. Timișoara, Romania) as a vehicle.

The mice were divided into four groups (*n* = 5), as follows: negative control (CONTROL), positive control (ASTHMA), preventive VD treatment group (PREV) and rescue VD treatment group (RESCUE). 

**Figure 7:**
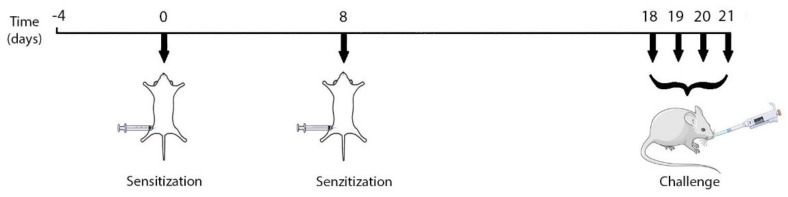
Timeline of mouse model induction. Graphical elements retrieved from https://smart.servier.com/ (accessed on 21 February 2021).

For the sensitization step, the groups ASTHMA, PREV and RESCUE were injected intraperitoneally (i.p.) with 1 mL of OVA-alum 50% solution, in two consecutive doses, seven days apart (Figure 7). For the challenge step, the mice in the lots above were instilled 12.5 µL of the 40% OVA solution in each nostril, using a calibrated micropipette, for four consecutive days, as described in Figure 7. The mice in the CONTROL group received only a sterile saline solution for both sensitization and challenge.

#### 4.1.3. Experimental Intervention: VD Treatment Regimen

**Figure 8:**
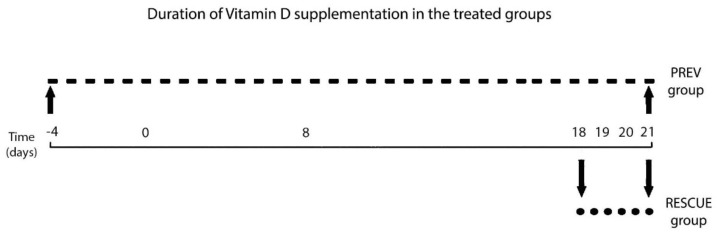
Timeline of VD treatment in the PREV and RESCUE groups.

The animals were treated with a commercially available (Vigantol Oil 0.5 mg/mL, Merck KGaA, Darmstadt, Germany) cholecalciferol (vitamin D3) solution via oral gavage, using a micropipette. The PREV group received a low-dose long-term supplementation regimen, considered a protective therapy: a daily dose of 50 µg/kg VD until the experiment ended (total dose of 1300 µg/kg). Therefore, mice in the PREV group received five doses of VD before the inflammation induction protocol was started (Figure 8). Mice in the RESCUE group were given a high-dose short-term supplementation regimen, described as salvation therapy: four consecutive daily doses of 100µg/kg VD, from days 18 to 21 (total dose of 400 µg/kg). Therefore, in the RESCUE group, supplementation was initiated only before the challenge step of the inflammation protocol (Figure 8). The ASTHMA and CONTROL group did not receive any treatment.

#### 4.1.4 Harvesting of Biological Samples

After completing the experiment, the animals were anaesthetized with 100 mg/kg/dose of ketamine and 10 mg/kg/dose of xylazine i.p. [101]. Blood was drawn by retro-orbital puncture and centrifuged immediately, with serum stored at −20 °C. A tissue incision was made to reveal the trachea, which was cannulated using a 20 G medical human venous catheter attached to a 1 mL syringe [102]. The lower airways and lungs were washed via a catheter with 0.8 mL of a phosphate-buffered saline solution (PBS, from Sigma-Aldrich Company, St. Louis, MO, USA) [103]. The aspirated BALF was immediately stored in aliquots placed on ice, followed by centrifugation, with the collection and storage of the supernatant fluid at −20 °C. Both lungs were harvested, with the left lateral lobe and right cranial and middle lobes placed in formaldehyde for pathology analysis. The remaining lung tissue was washed with ice-cold PBS, homogenized in a 1:1 solution of PBS and centrifuged, and the supernatant was collected and stored at −20 °C.

### 4.2. Assessment of Oxidative Stress

In this experiment, we evaluated the oxidative stress parameters in serum, BALF and LTH. 

TOS was determined following a colorimetric assay [45], and the results were expressed in μmol H_2_O_2_ equiv./L. This indirect method for reporting the oxidative stress level is based on the oxidation of ferrous ions to ferric ions determined by various reactive oxygen species in an acidic medium. 

TAR was investigated using a colorimetry method [63] and reported in μmol Trolox equiv./L. This assay is based on the inhibition of hydroxyl-radical-driven oxidative reactions, which are suppressed by the antioxidants present in the analyzed sample, a process followed by changes in the absorbance of colored dianisidyl radicals. 

OSI was calculated as TOS divided by TAR, with the ratio describing the oxidative stress level. 

Lipid peroxidation was evaluated by measuring MDA levels (nmol/mL) in biological samples, using the thiobarbituric acid method [104]. 

SH were measured using Ellman’s reagent [105]. The results were compared to a standard curve defined by GSH solution, ranging from 0.25 to 2 mM, with SH levels recorded as mmol GSH/mL [105]. 

All assays were performed utilizing a Jasco V-530 UV-Vis spectrophotometer (Jasco International Co. Ltd., Tokyo, Japan).

### 4.3. Assessment of Inflammation 

To assess the degree of Th2 inflammatory response, IL-4 was determined using the ELISA technique (Stat Fax 303 Plus Microstrip Reader, Minneapolis, MN, USA) with a commercially available kit (IL-4, Mouse IL-4 kit, MyBioSource, Inc, San Diego, CA, USA, Lot L191031878).

The anti-inflammatory effect of VD treatment was also assessed by measuring the inhibition of NO formation. The method estimated the degree of NO production through its final stable products (NOx), nitrite (NO_2_^-^) and nitrate (NO_3_^-^), using a Griess reaction to indirectly assess the NOx synthesis [106] in serum, BALF and LTH. The concentration of NOx was expressed as nitrite µmol/L. 

### 4.4. Pathology Analysis 

#### 4.4.1. Histopathology and Immunohistochemistry

For the histological analysis, three lung fragments were collected from the left lateral lobe and right cranial and middle lobes [107]. The tissues were fixed in 10% phosphate-buffered formalin for 24 h and routinely processed and embedded in paraffin wax. From each tissue fragment, two serial sections of 2–3 µm were stained with HE and PAS-AB. 

The histological changes were assessed in a semiquantitative manner [108] as: 0, normal; grade 1, few inflammatory cells; grade 2, a ring of inflammatory cells (one cell layer); grade 3, a ring of inflammatory cells composed of 2–4 layers, and grade 4, consisting of a ring of > 4 layers of inflammatory cells. The number of TB positive cells was quantified in five 20x fields [109]. 

#### 4.4.2. Immunohistochemical Analysis of NF-kB 

For the immunohistochemical analysis of NF-kB, the sections were incubated at 37 °C for 12 h and processed using the automatic platform Leica BOND-MAX (Leica Biosystems, Buffalo Grove, IL, USA). The primary polyclonal antibody anti-NF-kB p65 (PA5-27617, lot VC2970145, Invitrogen, Thermo Fisher Scientific, Waltham, MA, USA) was diluted in 1% PBS-BSA (bovine serum albumin) at 1:500. The Bond Polymer Refine Detection kit (DS9800, Novocastra, Leica Biosystems, Buffalo Grove, IL, USA) containing a peroxide block, post-primary polymer reagent, DAB chromogen and hematoxylin counterstain was used. For the negative control, the primary antibody was replaced by a normal rabbit IgG.

The positive reaction was given by the brown labeling of the cells, in either or both the cytoplasm and the nucleus. NF-kB p65 immunopositivity was firstly evaluated via the number of positive cells (grade 0, none; grade 1, <1/3 of stained cells; grade 2, between 1/3–2/3 of stained cells; grade 3, >2/3 of stained cells) and secondly, via intensity (0, negative; 1, between 0–2; 2, strong) [110,111].

Two pathologists (AU and MT) independently examined the sections using a light Olympus BX-41 microscope (Shinjuku, Tokyo, Japan). When there was a divergence of opinions, an agreed diagnosis was reached by a simultaneous evaluation in a multi-head microscope Zeiss Axio Scope A1 (Carl Zeiss Microscopy GmbH, Germany). The photomicrographs were taken using an Axiocam 208 color digital camera and ZEN core imaging software (Carl Zeiss AG, Oberkochen, Baden-Württemberg, Germany). 

### 4.5. Statistical Analysis 

A statistical analysis was conducted using SPSS version 20.0.0 (IBM, Armonk, NY, USA). Numerical data are presented as the mean ± standard deviation. When appropriate, the distribution of results was plotted as individual values and median. The data were evaluated for normality with the aid of the Shapiro–Wilk test, and the variances were tested using Levene’s test; subsequently, the means were compared using the appropriate independent t-test [112], and the corresponding *p* values were reported. For non-normal distributed data, a Mann–Whitney U test was performed, with outcomes presented as the median and interquartile range (IQR) with the corresponding *p* values.

## 5. Conclusions

Oral supplementation with VD seems to play a protective role in balancing the oxidative stress associated with asthmatic inflammation. VD reduced the oxidative components and increased the antioxidant capacity in the serum and LTH of treated mice. We did not observe a statistically significant difference between long-term low-dose and short-term high-dose VD therapy. 

## Data Availability

The data are not publicly available until an associated PhD thesis is published. The data presented in this study will be available on request from the corresponding author.
